# Connecting the dots: Stopover strategies of an intercontinental migratory songbird in the context of the annual cycle

**DOI:** 10.1002/ece3.3227

**Published:** 2017-07-21

**Authors:** Kristina L. Paxton, Frank R. Moore

**Affiliations:** ^1^ Department of Biological Sciences University of Southern Mississippi Hattiesburg MS USA; ^2^ Present address: Department of Biology University of Hawaii Hilo Hilo HI USA

**Keywords:** black‐and‐white warblers, full annual cycle, *Mniotilta varia*, seasonal interactions, stable isotopes

## Abstract

The phases of the annual cycle for migratory species are inextricably linked. Yet, less than five percent of ecological studies examine seasonal interactions. In this study, we utilized stable hydrogen isotopes to geographically link individual black‐and‐white warblers (*Mniotilta varia)* captured during spring migration with breeding destinations to understand a migrant's stopover strategy in the context of other phases of the annual cycle. We found that stopover strategy is not only a function of a bird's current energetic state, but also the distance remaining to breeding destination and a bird's time‐schedule, which has previously been linked to habitat conditions experienced in the preceding phase of the annual cycle. Birds in close proximity to their breeding destination accumulate additional energy reserves prior to arrival on the breeding grounds, as reflected by higher migratory condition upon arrival, higher refueling rates measured via blood plasma metabolites, and longer stopover durations compared to birds migrating to breeding destinations farther from the stopover site. However, late birds near their breeding destination were more likely to depart on the day of arrival (i.e., transients), and among birds that stopped over at the site, the average duration of stopover was almost half the time of early conspecifics, suggesting late birds are trying to catch‐up with the overall time‐schedule of migration for optimal arrival time on the breeding grounds. In contrast, birds with long distances remaining to breeding destinations were more likely to depart on the day of arrival and primarily used stopover to rest before quickly resuming migration, adopting similar strategies regardless of a bird's time‐schedule. Our study demonstrates that migrants adjust their en route strategies in relation to their time‐schedule and distance remaining to their breeding destination, highlighting that strategies of migration should be examined in the context of other phases of the annual cycle.

## 1 INTRODUCTION

Although the phases of the annual cycle for migratory species are temporally and geographically disparate, there is a growing recognition of seasonal interactions and the interconnectedness of different phases of the annual cycle (Harrison, Blount, Inger, Norris, & Bearhop, 2010). If we are to understand the biology of a migratory species in any one phase of the annual cycle, we must know how different phases interact and connect with one another (Marra, Cohen, Loss, Rutter, & Tonra, 2015). Yet, less than five percent of ecological studies examine seasonal interactions between phases of the annual cycle (Marra et al., 2015). Studies that consider seasonal interactions primarily focus on linking stationary phases (e.g., McKellar, Marra, Hannon, Studds, & Ratcliffe, 2013; Reudink et al., 2009; Saino et al., 2004) and largely ignore the intervening migratory period (but see Bearhop, Hilton, Votier, & Waldron, 2004; Yohannes, Lee, Popenko, & Bauchinger, 2012; Paxton, Cohen, Paxton, Nemeth, & Moore, 2014) where mortality is thought to be substantial (Newton, 2008; Paxton, Durst, Sogge, Koronkiewicz, & Paxton, 2017; Sillett & Holmes, 2002). However, 30% or more of a migrant's annual cycle is spent on migration, with over 70% of that time spent at stopover sites (Hedenstrom & Alerstam, 1997) where energy expenditure is high relative to migratory flight (Wikelski et al., 2003). Whereas there is a rich body of literature on the strategies of migration (reviewed in Newton, 2008), until the en route phase is incorporated into a full annual cycle approach we will not achieve a comprehensive understanding of migration (Moore, Smith, & Sandberg, 2005).

The success of migration is largely determined by the rate at which birds refuel at a stopover site and the duration of their stopover, two critical determinants of the time spent on migration (e.g., time‐minimization sensu Alerstam & Lindstrom, 1990; Hedenstrom, 2008). That said, a migrant's stopover strategy (i.e., refueling rate and duration of stopover) is not only a function of a bird's current energetic state, quality of stopover habitat, and weather conditions (Moore, Gauthreaux, Kerlinger, & Simons, 1995), it is also influenced by the cumulative effect of conditions encountered prior to arrival at a stopover site as well as the distance remaining to destination. Despite the intuitive appeal of this argument, empirical evidence is limited for small landbird migrants because of the challenges in following migrants throughout their annual cycle.

Advances in intrinsic (e.g., stable isotopes, genetic markers; Hobson & Wassenaar, 2008; Ruegg et al., 2017) and extrinsic (e.g., light‐weight tracking devices; Bridge et al., 2011) markers to track and model animal movement have made it increasingly possible to place migration in the context of other periods of the annual cycle, enhancing our understanding of how migrants are spatially (Bairlein et al., 2012; Ruegg et al., 2014) and temporally (Kelly, 2006; Paxton, van Riper, THeimer & Paxton, 2007; Stutchbury et al., 2011) distributed during migration. Further, growing evidence of seasonal interactions has revealed that the quality of winter habitat influences en route timing (Paxton & Moore, 2015) and timing of arrival on the breeding grounds (Marra, Hobson, & Holmes, 1998) with cascading impacts on reproductive performance (Norris, Marra, Kyser, Sherry, & Ratcliffe, 2004; Reudink et al., 2009). However, a migrant's condition during migration is only diffusely related to a bird's winter habitat quality (González‐Prieto & Hobson, 2013; McKinnon, Stanley, & Stutchbury, 2015; Paxton & Moore, 2015; but see Bearhop et al., 2004), suggesting that winter habitat quality only sets the stage for spring migration. Given the importance of both timing and condition upon arrival to the breeding grounds on reproductive success (Moller, 1994; Moore, Smith, et al., 2005; Norris et al., 2004), it is essential to understand how migrants adjust migration strategies in relation to other phases of the annual cycle to optimize migration performance, measured in terms of time and condition.

In this study, we utilize stable hydrogen isotopes (δ^2^H) to geographically link individual black‐and‐white warblers (*Mniotilta varia)* captured during spring stopover with breeding destinations to examine a migrant's stopover strategy (refueling rate and stopover duration) in the context of other periods of the annual cycle. In doing so, we account for population‐specific differences in the timing of migration between geographically diverse populations of black‐and‐white warblers. Specifically, we determine (1) the proximity of an individual to their breeding destination (i.e., short or long distances remaining) and (2) whether an individual is early or late relative to other birds migrating to the same breeding destination. We have previously shown that the quality of winter habitat strongly influences timing of arrival at stopover (Paxton & Moore, 2015). Among black‐and‐white warblers migrating to the same breeding destination, individuals from poor‐quality winter habitat arrive later to the stopover site than birds originating from high‐quality winter habitat (Paxton & Moore, 2015). In this study, we can now examine how the consequences of late arrival to stopover sites due to winter habitat quality influences the strategies utilized en route.

Integrating information about an individual's breeding destination and timing of migration relative to conspecifics allows us to examine stopover strategies in the context of other phases of the annual cycle. Our study focuses on two strategies critical to migration success, refueling rates and stopover duration (Alerstam & Lindstrom, 1990). We utilize blood plasma metabolites as a robust measure of refueling performance during stopover (Guglielmo, Cerasale, & Eldermire, 2005; Seewagen, Guglielmo, & Morbey, 2013), and a mark‐recapture framework to estimate stopover duration of black‐and‐white warblers (Pradel, Schaub, Lukas, & Lebreton, 2005; Schaub, Pradel, Jenni, & Lebreton, 2001). We hypothesize that an individual's time‐schedule during migration and the distance remaining to breeding destination will modulate an individual's strategy at stopover sites. Specifically, we predict that individuals arriving late to the stopover site will try to “catch‐up” with the overall time‐schedule of migration (sensu Alerstam & Lindstrom, 1990) by (1) refueling at a faster rate and (2) staying for a shorter duration than conspecifics migrating to the same breeding destination. However, we also predict that the distance remaining to a bird's breeding destination will influence stopover strategies with individuals migrating longer distances to more northern breeding destinations having (1) higher refueling rates and (2) shorter durations of stopover in order to attain a higher speed of migration than individuals migrating shorter distances to southern breeding destinations (sensu Alerstam & Lindstrom, 1990; see also Ellegren, 1993; Frannson, 1995).

## 2 MATERIALS AND METHODS

### 2.1 Ethics statement

The research conducted for this study was carried out in accordance with the Ornithological Council's guidelines for the use of wild birds in research and was approved by The University of Southern Mississippi's institutional animal care and use committee (protocol #1092210). Other permits were from the United States Department of the Interior bird banding laboratory (permit #21221) and Fish and Wildlife Service (permit #MB75836‐3) and the Louisiana Department of Wildlife and Fisheries (permit #LNHP‐11‐058).

### 2.2 Study site and species

We examined stopover strategies at a long‐term migration station located approximately 1.5 km inland from the Gulf of Mexico within a narrow, isolated coastal woodland in Cameron Parish (29˚45′N, 93˚37′W), Louisiana, USA. The landscape in southwestern Louisiana is dominated by open grassy marsh and wet prairie with forest occurring on narrow and elongated coastal ridges called Cheniers (e.g. Moore, 1999). Trans‐gulf migrants generally fly over the coastal plain in southwestern Louisiana during spring passage and make landfall in more contiguous forest cover ˃50 km inland (Gauthreaux, 1971). However, coastal Cheniers often concentrate migrants because they are islands of suitable forested habitat, surrounded by unsuitable stopover habitat, and are especially attractive to migrants during weather conditions unfavorable for northward movement (Gauthreaux, 1971) or when energetically stressed (e.g., Yong & Moore, 1997). While food resources to replenish fat and muscle stores within coastal Cheniers are often lower than in larger more contiguous hardwood forests further inland (Buler, Moore, & Woltmann, 2007), many migrant species, including black‐and‐white warblers, replenish fuel stores during multiple day stopovers in coastal woodlands (see Moore, Woodrey, Buler, Woltmann, & Simons, 2005; Yong & Moore, 1997). Examining migration strategies of multiple breeding populations utilizing a single stopover site removes the confounding effect of habitat and resource availability that can differ among multiple stopover sites.

We captured black‐and‐white warblers during spring migration from 2008 to 2011 with mist‐nets (12 × 2.6 m, 30 mm mesh) on a daily basis, 21 March to 13 May, for approximately 8 hr (0800–1700). Migrants typically begin to arrive on the northern Gulf coast after nonstop flight across the Gulf of Mexico in the late morning (10:00–14:00; Gauthreaux, 1971). Less than 10% of black‐and‐white warblers included in this study were captured before 11:00, suggesting that the majority of captured warblers were newly arriving migrants to the stopover site. Black‐and‐white warblers do not winter or breed in southeastern Louisiana; so all warblers captured were migrants. Nets were monitored every 30 min, and birds were brought to a central location for blood sampling and banding. We recorded the time of day and time elapsed between extracting a bird from a net and blood sampling to examine the potential influence of these factors on plasma metabolite values (see 2.8). All birds were bled within 20 min of capture, with blood samples taken on average within 6.03 ± 0.34 min after extracting a warbler from a mist net. Upon capture, we banded black‐and‐white warblers with a USGS band and a unique color band combination, and took a blood sample via brachial vein puncture for blood plasma metabolites (plasma) and stable carbon isotope (δ^13^C) analysis (red blood cells used in Paxton & Moore, 2015). After blood sampling, we aged (after‐second year or second year) and sexed birds according to Pyle (1997), and then weighed birds to the nearest 0.1 g with an electronic scale. Additionally, we pulled two tail feathers (R5) for δ^2^H analysis. We stored blood samples on ice, and then separated plasma and red blood cells, freezing samples at −20°C within 6 hr of collection.

### 2.3 Stable isotope analysis

We cleaned feathers with a dilute detergent and then a 2:1 chloroform:methanol solution following the method of Paritte and Kelly (2009). Feather material from the distal end (140–160 ug) of each sample was removed and wrapped in a silver capsule. We conducted all stable isotope analyzes at the University of Oklahoma with a ThermoFinnigan Delta V isotope‐ratio mass spectrometer connected to a high‐temperature pyrolysis elemental analyzer (TC/EA, ThermoFinnigan, Bremen, Germany). We utilized a comparative equilibrium approach with calibrated keratin standards to correct for uncontrolled isotope exchange between non‐carbon‐bound hydrogen in feathers and ambient water vapor (Wassenaar & Hobson, 2003). Stable hydrogen isotope ratios (^2^H/^1^H) are reported in delta (δ) notation, in per‐mil units (‰), where δ^2^H_sample_ = [(*R*_sample_/*R*_standard_)−1] × 1,000, relative to a standard (Vienna standard mean ocean water [VSMOW]). Further details about our isotope measurement methods can be found in Paxton and Moore (2015).

### 2.4 Breeding destination

**Figure 1:**
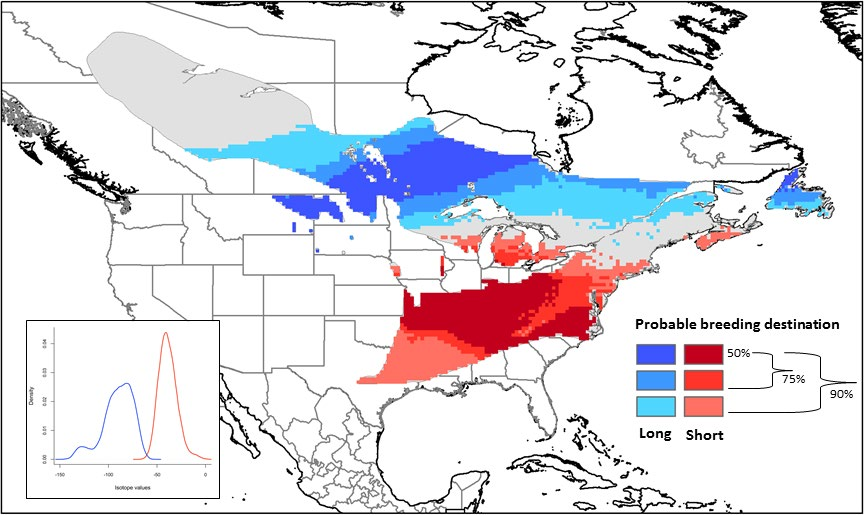
Probable breeding destination of black‐and‐white warblers sampled during spring migration classified as short distance migrants in the southeastern U.S. (red colors, δ^2^H values between −10 and −50‰) and long distance migrants in the boreal forest of Canada (blue colors, δ^2^H values between −70 and −130‰). Based on the actual δ^2^H value of warblers classified into each breeding group, plus estimated error, the probable breeding destination of 50% of warblers is contained within the darkest colored areas, 75% within the medium and dark colored areas, and 90% within all color shades. Insert depicts the probability distribution of isotope values (incorporating associated error) of warbles sampled during migration color coded by breeding group

The use of stable isotopes to infer breeding destinations of migrants during the nonbreeding time period has been widely applied to many Neartic‐Neotropical migrants that molt their feathers on the breeding ground prior to migration (reviewed in Hobson & Wassenaar, 2008), including the black‐and‐white warbler (Hobson et al., 2014; Marra & Studds, 2013). However, individual differences in physiology, analytical error, and error associated with the isoscape to which birds are assigned precludes using δ^2^H values of feathers alone to directly assign individuals to specific geographic locations (Wunder & Norris, 2008). Therefore, we calculated the probability that an individual black‐and‐white warbler captured at the migration station was associated with a breeding destination, utilizing information from both the feather sample and error associated with stable hydrogen isotope values of feathers (δ^2^H_f_) and precipitation (δ^2^H_p_; Fig. 1). We selected two breeding destinations that differed in their distance from the stopover site: southeastern U.S. (short distances, 125–1,750 km) and boreal forest of Canada (long distances, 2,000–3,000 km). By defining two breeding destinations with a range of δ^2^H_p_ values (southeast: −10 to −50‰, boreal forest: −70 to −130‰) that do not overlap in probability of breeding destinations when taking into account error (Fig. 1), we have a high certainty that black‐and‐white warblers within these two groups are migrating to separate breeding destinations (i.e., southeast or boreal forest). While it is assumed that birds are migrating to the same breeding region as the previous breeding season based on high site fidelity of adults (Kricher, 2014), there is no information on the distance of natal dispersal. However, defining breeding destinations across a broad range increases the likelihood that birds dispersing to new locations will remain in the same breeding region.

We adjusted δ^2^H_f_ of warblers captured at the stopover site to reflect δ^2^H_p_ utilizing the equation: δ^2^H_f_ = 0.95 (δ^2^H_p_)−17.57 (nonground foraging Nearctic‐Neotropical migrants; Hobson, Van Wilgenburg, Wassenaar, & Larson, 2012). To visualize the likely breeding destination for the ranges of δ^2^H_p_ values specified above, similar to Hobson, Lormée, Van Wilgenburg, Wassenaar, and Boutin (2009), we utilized Program R to generate a random sample of 1,000 possible isoscape values for each isotope value sampled using a normal distribution with the isotope values as the mean and the average error (5.5‰ *SD*; error calculation described in Paxton & Moore, 2015). We derived a probability density function from the simulated isotope values, allowing us to estimate the probability of breeding destination of individuals assigned to each of the two breeding destinations, calculated for all possible δ^2^H_p_ values in black‐and‐white warblers’ breeding range. Using the calculated probability densities, we then reclassified the Bowen, Wassenaar, and Hobson (2005) isoscape in ArcGIS ver. 10.0 (ESRI, Redlands, CA) to indicate the geographic range of warblers included for each breeding destination (southeast and boreal forest), in 50%, 75%, and 90% probability of breeding destination categories (Fig. 1).

### 2.5 Timing of migration and migratory condition

Because of significant differences among years, males and females, and population‐level differences in the timing of arrival to the stopover site (*F*_5,155_ = 28.17, *p* < .001; Appendix 1), we divided each migration season (2008, 2009, 2010, 2011) into three equal time periods (early, middle, late) based on the range of capture dates for males and females in each black‐and‐white warbler breeding destination (southeast and boreal forest) in a given year. This allowed us to specifically examine migration strategies of early and late migrants without the confounding effects of: (1) year‐to‐year variability in timing of arrival to the stopover site, and (2) population‐specific differences in the timing of migration between geographically diverse populations of black‐and‐white warblers. Therefore, comparisons of refueling and stopover duration between early and late migrants are relative to conspecifics of the same sex that were migrating to the same breeding destination.

To determine the condition of a black‐and‐white warbler captured at the stopover site, we subtracted a size‐specific fat‐free mass from actual body mass at capture (Owen & Moore, 2006). The equation fat‐free mass = 0.014*wing cord + 2.0086 was used to estimate size‐specific fat‐free mass for each black‐and‐white warbler captured at the stopover site based on the relationship between fat‐free mass and specific wing cord classes of black‐and‐white warblers captured in previous years at our study site (1993–2007, *n* = 684; described in Paxton & Moore, 2015). Larger migratory condition values indicate birds with more fat stores, and thus better migratory condition.

### 2.6 Measurement of refueling rate

Measurement of blood plasma metabolites is a robust method to assess the short‐term refueling rate (30–120 min) of an individual bird based on a single capture (Jenni‐Eiermann & Jenni, 1994; Schaub & Jenni, 2001; Zajac, Cerasale, & Guglielmo, 2006), and has been widely used as a relative measure of refueling performance during stopover (Cerasale & Guglielmo, 2010; Guglielmo et al., 2005; Seewagen et al., 2013). Plasma triglyceride levels increase during feeding and fat deposition, whereas β‐hydroxy‐butyrate levels increase during fasting and mass loss, with both metabolites reflecting subtle changes in body mass (Jenni‐Eiermann & Jenni, 1994; Zajac et al., 2006). Given that migrants begin to explore and forage at a stopover site almost immediately after nonstop flight across the Gulf of Mexico (Moore, Kerlinger, & Simons, 1990), metabolite concentrations provide a sensitive index of refueling rates for black‐and‐white warblers captured at the stopover site. Prior to laboratory analysis, we diluted all plasma samples twofold in 0.9% NaCl solution so that concentrations of samples fell within the standard curve. Metabolites were assayed on a microplate spectrophotometer in 400‐ml flat bottom microplates. We measured plasma triglyceride sequentially by colorimetric endpoint assay (Sigma, St. Louis, MO: 5 μl plasma, 240 μl glycerol reagent, and 60 μl triglyceride reagent; Guglielmo, OHara, & Williams, 2002), calculating plasma triglyceride concentrations by subtracting free glycerol from total glycerol. We measured β‐hydroxy‐butyrate directly by kinetic endpoint assay (R‐Biopharm, Marshall, MI, USA; Guglielmo et al., 2005). For each assay, samples were analyzed in duplicate, and values were averaged. All coefficients of variation between replicate samples were less than 15%.

### 2.7 Food availability

To examine if food availability changed within and among years, we sampled arthropod abundance via branch clipping (Cooper & Whitmore, 1990) once a week during each migration season. We collected five branch samples per session approximately 1.5–2 m above the ground from each of the three dominant tree species at the site: live oak (*Quercus virginiana*), honey locust (*Gleditsia triacanthos*), and hackberry (*Celtis occidentalis*). The arthropod sampling was designed to provide a broad representation of food availability for migrants utilizing the site, and encompasses the foraging locations (e.g., mid canopy) and diet (e.g., Lepidoptera larvae, Hymenoptera, Coleoptera) of black‐and‐white warblers (Kricher, 2014). While black‐and‐white warblers often feed along the bark of trunks and limbs, which is not sampled with this design, they have a wide foraging niche that also includes foliage gleaning at mid canopy similar to other wood‐warblers (Kricher, 2014). We identified all arthropods collected to order and measured their length. Dried vegetation material from each clipping was weighed with an electronic scale. Arthropods abundance is presented as the number of arthropods observed per gram of vegetation per sampling session.

### 2.8 Statistical analysis

Metabolite concentrations were ln + 1 transformed to satisfy assumptions of normality. We assessed the strength of the relationship between plasma triglycerides and β‐hydroxy‐butyrate with a correlation analysis, and calculate a refueling index for each warbler based on the first principal component axis of a principal component analysis (PCA) of transformed concentrations of plasma triglycerides and β‐hydroxy‐butyrate (total *n* = 103, including black‐and‐white warblers not assigned to the two breeding destinations (*n* = 25); Schaub & Jenni, 2001; McGuire, Fenton, & Guglielmo, 2009; Cerasale & Guglielmo, 2010). Larger refueling index values indicate higher refueling rates; however, the average refueling index scores is zero, hence negative scores do not reflect negative refueling rates, but refueling index scores below the average value.

We examined variation in refueling index values with a two‐tiered modeling approach. First, we selected a priori four methodological variables that have been shown to influence plasma metabolite levels for model inclusion (Guglielmo et al., 2002, 2005): time in minutes elapsed between extracting a bird from a mist net and blood sampling (Bleedtime), time in minutes elapsed between sunrise and blood sampling (Daytime), the day of the year when the blood sample was collected (Season), and year blood sample was taken (Year). Assumptions of multicolinearity where not violated; however, to satisfy assumptions of normality we ln + 1 transformed Bleedtime. We constructed general linear models with refueling index values as our response variable and all possible combinations of each factor (*n* = 15) and a null model using R package MuMIn (Barton, 2014). We used Akaike's information criterion adjusted for small sample sizes (AICc; Burnham & Anderson, 2002) to rank candidate models. Variables contained in the top model were then included in all subsequent refueling models.

In our second set of candidate models, we constructed general linear models to examine variation in refueling index values as a function of a warbler's breeding destination categorized as southeast or boreal forest (Breeding) and timing of migration relative to conspecifics migrating to the same breeding destination categorized as early, middle, or late (Timing). In addition, we included a warbler's migratory condition (Condition), the age of the warbler categorized as second year or after‐second year (Age), and sex of the warbler (Sex) as factors in the models given the importance of these variables in refueling rates during stopover (Moore et al., 1995). We included the methodological variable, Bleedtime, in all models given support of its influence on refueling index values from the first model selection procedure. To evaluate the importance of each explanatory variable, candidate models (*n* = 144) included a fully specified global model with all main effects, a null model, and reduced forms of the general model including all possible combinations of each factor (using package MuMIn in R; Barton, 2014). We incorporated biologically meaningful second‐order interactions in both the full general model and reduced forms. Biological meaningful second‐order interactions included the following: breeding with timing, age, and sex, and timing with age and sex. We used AICc to rank, compare, and evaluate all candidate models. We present all models with ΔAICc ≤ 4 as possible competing models (considered the subset of best models; Burnham & Anderson, 2002), and also present the null model for assessing the relative explanatory power of the models under consideration. For explanatory variables in top models, we averaged parameter estimates across models containing each explanatory variable and calculated standard errors from unconditional variances due to model selection uncertainty (Burnham & Anderson, 2002; R package “MuMIn”; Barton, 2014). We also estimated the relative importance of each variable (*j*) by calculating *w *+ (*j*), where *w *+ (*j*) is the sum of *w*_*i*_ (Akaike weights) across all models in the set in which variable *j* occurred (Burnham & Anderson, 2002). Variables with strong support have cumulative Akaike weights near 1.

We accounted for imperfect detection of warblers that stopover at the stopover site using a mark‐recapture framework to estimate stopover duration of black‐and‐white warblers (Schaub et al., 2001), using both recaptures and resights subsequent to initial capture. We systematically searched for color‐banded black‐and‐white warblers using transects spaced throughout the study site on a daily basis in 2009, 2010, and 2011. We used Program MARK (White & Burnham, 1999) to produce daily survival estimates using a Cormack–Jolly–Seber model (CJS), separating first and subsequent encounters to account for transients (i.e., birds that do not stay at the stopover site beyond the day of arrival; Pradel et al., 2005). With an assumption of no mortality at the stopover site, these daily survival rates are equivalent to an average daily stopover rate, with stopover duration calculated in the same manner as an expected lifespan (Schaub et al., 2001). We were not interested in changes in stopover duration over the season, and therefore collapsed the encounter history so that the initial capture of all birds was “day 1,” and the total encounter history file was nine periods, the longest span between detections of any one individual. No resights were conducted in the first year of the study, and we used a year function (1st year vs. all others) to account for a different detection probability in the first year. We examined the relative effects of timing, breeding, age, sex, and migratory condition on stopover duration using AICc. We model averaged parameter estimates from the top models (AICc ≤ 4), and calculated the average expected stopover duration of nontransients based on their timing, breeding destination, sex, and migratory condition. To estimate the percentage of individuals that are transient for each group (and average condition), we divided the average daily stopover (i.e., survival) probability of all birds in the period immediately following initial capture by the average daily stopover of individuals thereafter (i.e., nontransients; Salewski & Schaub, 2007), with variance approximated using the delta method.

We assessed changes in arthropod abundances within a season and among years using regression and one‐way ANOVA analysis, respectively. We performed all statistical analyzes in R version 3.3.2 (R Development Core Team 2016), and MARK 7.1 (White & Burnham, 1999).

## 3 RESULTS

**Figure 2:**
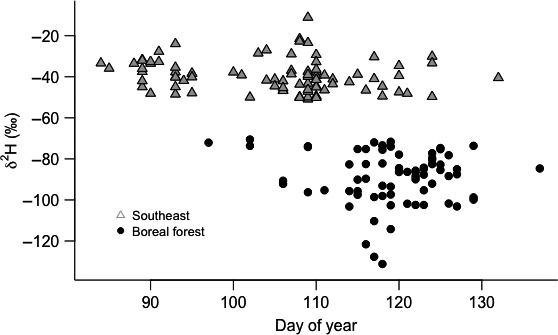
Distribution of stable hydrogen isotope values (δ^2^H) of black‐and‐white warblers captured during spring migration 2008–2011 on the northern Gulf of Mexico. Within each breeding destination, southeastern U.S. and boreal forest of Canada, there was not a significant relationship between δ^2^H and the day of the year when a black‐and‐white warbler was captured, indicating that warblers captured late where not migrating to breeding destinations at more northern latitudes than earlier arriving conspecifics

During the four‐year study period, we captured a total of 223 black‐and‐white warblers, with warblers migrating to breeding destinations in the southeastern U.S. (*n* = 88) and boreal forest of Canada (*n* = 73) representing three quarters of the warblers utilizing the stopover site (Table 1). Males ($\overset{¯}{x}$ day of year = 110.9 ± 0.97) and birds migrating to breeding destinations in the southeastern U.S. ($\overset{¯}{x}$ day of year = 105.0 ± 1.1) arrived before females ($\overset{¯}{x}$ day of year = 113.91 ± 0.97) and birds migrating to breeding destinations in the boreal forest of Canada ($\overset{¯}{x}$ day of year = 119.11 ± 0.08). However, within each breeding group, there was no relationship between the day of year when a bird was captured and δ^2^H (southeast: *F*_1,86_ = 2.70, *p* = .10, *R* ^2^ = .02, boreal: *F*_1,71_ = 0.44, *p* = .51, *R* ^2^ = .01; Fig. 2), indicating that birds arriving late to the stopover site were not migrating to more northern breeding destinations than earlier arriving conspecifics.

**Table 1:** Number of black‐and‐white warblers captured during spring migration 2008–2011 on the northern Gulf of Mexico assigned to breeding destinations in the southeastern U.S. and boreal forest of Canada (*n* = 161). We examined the range of capture dates for male (M) and female (F) black‐and‐white warblers assigned to each breeding destination separately, dividing each migration season into three equal time periods (early, middle, late) based on the range of capture dates for males and females in each breeding destination in a given year. We successfully assayed triglyceride and β‐hydroxy‐butyrate concentrations for a subset of the warblers (*n* = 85), indicated by the number in the parentheses

Migration timing	Breeding destination	
	Southeast M/F	Boreal forest M/F
Early	10/17 (5/8)	20/10 (10/5)
Middle	13/21 (5/7)	13/11 (10/6)
Late	12/15 (9/9)	11/8 (6/5)

### 3.1 Refueling rate

Similar to plasma metabolite levels of migrants at other stopover sites in North America (Cerasale & Guglielmo, 2010; Guglielmo et al., 2005), average plasma triglyceride and β‐hydroxy‐butyrate concentrations for black‐and‐white warblers were 2.22 mmol/L (range: 0.19–8.55 mmol/L) and 1.61 mmol/L (range: 0.21–5.00 mmol/L), respectively. There was a negative correlation between plasma concentrations of transformed triglycerides and β‐hydroxy‐butyrate (*r* = −0.46, *t* = −5.26, *df* = 101, *p* < .001), and based on a PCA analysis, the first principal component axis explained 76% of the variation between the two metabolites. Triglyceride concentrations loaded positively (+0.88) and β‐hydroxy‐butyrate concentrations negatively (−0.48) along the first principal component axis. Thus, the first principal component score (i.e., refueling index) positively correlates with refueling rates.

Model selection of methodological variables suggested that time between extracting a bird from the mist net and blood sampling (Bleedtime) most strongly influenced refueling index values (Table 2). We did not find strong support for the influence of year, time of day, or day of year on refueling index values. To control for the negative impact of the elapsed time between capture and blood collection on refueling (lnBleedtime: β = −0.21 ± 0.11) we included the variable bleedtime in all models examining variation in refueling index values.

**Table 2:** Summary of model results for variables influencing refueling index values (methodological and overall model results) and stopover duration of black‐and‐white warblers captured during spring migration 2008–2011 on the northern Gulf of Mexico. For overall refueling index and stopover duration models all top competing models (ΔAICc < 4) based on Akaike's information criterion adjusted for small sample sizes (AICc) and the null model are shown. Only the top model and null model are shown for the methodological refueling index model which examined how refueling index values are influenced by the time elapsed between capture and blood sample (Bleedtime), time elapsed between sunrise and blood sampling (Daytime), the day of the year when the blood sample was taken (Season), and the year of blood sample. Explanatory variables examined in the overall refueling index and stopover duration models include the following: timing of migration relative to conspecifics migrating to the same breeding destination (Timing), breeding destination in either the southeastern U.S. or boreal forest (Breeding), migratory condition of a bird at capture (Condition), age and sex of the warbler. We also included Bleedtime in all overall refueling index models based on the results of refueling index methodological analysis. For each model the Akaike weights (*w*_*i*_), deviance, and degrees of freedom (*df*) are shown

Model description^a^	*df*	Deviance	ΔAIC_c_ c	*w*_*i*_
Refueling index methodological				
Bleedtime	3	16.37	0.00	0.17
Null	2	20.11	6.44	0.00
Overall refueling index				
Breeding, timing, breeding*timing, condition, bleedtime	9	12.67	0.00	0.32
Breeding, timing, breeding*timing, condition, sex, bleedtime	10	12.52	1.55	0.15
Timing, condition, bleedtime	6	14.24	2.61	0.08
Breeding, timing, breeding*timing, bleedtime	8	13.56	3.29	0.06
Null	2	18.11	14.11	0.00
Stopover duration^b^				
Breeding, condition	20	1,916.15	0.00	0.39
Condition	19	1,918.71	0.49	0.31
Sex, condition	20	1,918.02	1.87	0.15
Timing, condition	22	1,915.22	3.23	0.08
Null	2	1,981.06	43.09	0.00

a Refueling index methodological and overall refueling index model were calculated using program R. Stopover duration model results calculated with the program MARK.

b Model variables only shown for survival a(phi). Detection probability (*p*) was the same for all top models *p*(breeding, timing, condition, year).

c AIC of top model: refueling index methodological: 107.51, overall refueling index: 99.84, stopover duration: 1,956.92.

**Figure 3:**
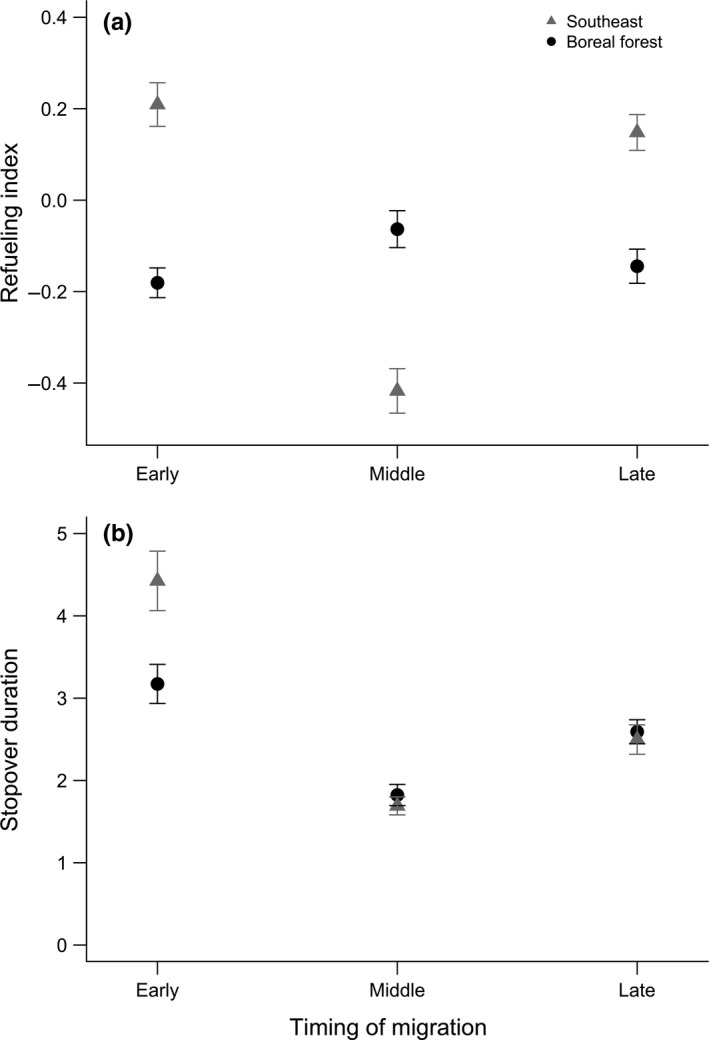
Average (a) predicted refueling index values (±*SE*) and (b) stopover duration estimates (±*SE*) for black‐and‐white warblers captured during spring migration 2008–2011 on the northern Gulf of Mexico. The migration season was divide into three equal time periods (early, middle, late) based on the range of capture dates for males and females in each breeding destination in a given year. Predicted refueling index values and stopover duration estimates were calculated from modeled average parameter estimates of top models (ΔAICc < 4). Refueling index values are a relative estimate of fuel deposition rates based on a principal component analysis of plasma triglycerides and β‐hydroxy‐butyrate concentrations. Larger refueling index values indicate higher refueling rates; however, the average refueling index scores is zero, hence negative scores do not reflect negative refueling rates, but refueling rates below the average value. Stopover duration estimates are for nontransient migrants only

A black‐and‐white warbler's breeding destination and the timing of arrival at the stopover site had strong support for influencing refueling index values (Table 2; relative importance (RI) breeding = 0.86, RI timing = 1.00). However, model selection also indicated strong support for an interaction between timing of migration and breeding destination (Table 2; RI = 0.86), suggesting different stopover strategies for refueling rates between early, middle, and late warblers migrating to breeding destinations in the southeastern U.S. and the boreal forest of Canada (Fig. 3a). The overall higher refueling index values for warblers migrating to breeding destinations in the southeast ($\overset{¯}{x}=0.009\;\pm \;0.09$) were driven by high refueling index values of warblers arriving early and late to the stopover site, and contrasted with the low refueling index values of birds arriving during mid migration. In contrast, refueling index values were consistently low among warblers migrating to the boreal forest ($\overset{¯}{x}=-0.13\;\pm \;0.05$), regardless of when during migration they stopped at the stopover site. There was also strong support for a positive influence of a warbler's migratory condition upon arrival to the stopover site on refueling index values (Table 2; RI = 0.90; β = 0.11 ± 0.06). There was very little support for males having higher refueling index values than females based on the relative importance of sex in top models and the overlap of the 95% confidence interval with zero (Table 2; RI = 0.24; β = 0.03 ± 0.07).

### 3.2 Food availability

Arthropod abundance on the three dominant plant species at the stopover site did not significantly differ among the 4 years of the study (*F*_3,18_ = 0.13, *p* = .94) with a mean of 0.15 ± 0.2 arthropods/g vegetation across all years (range: 0.03–0.41). Within a spring season, we also found no relationship between arthropod abundance and the day of the year (*F*_1,20_ = 0.05, *p* = .82, *R* ^2^ = .05).

### 3.3 Stopover duration

Forty‐two of the 161 (26%) black‐and‐white warblers migrating to breeding destinations in the southeastern U.S. and boreal forest of Canada were encountered at the stopover site beyond the day of capture (recaptures: *n* = 30, resighted: *n* = 33). The probability of a warbler departing the day of arrival (i.e., transient) was approximately 50% higher for warblers migrating long distances to the boreal forest compared to warblers migrating short distances to breeding destinations in the southeastern U.S. (Table 3). While there is some uncertainty in probability estimates of transients for timing and sex categories due to larger error estimates; among warblers migrating to the same breeding destination, warblers that arrived late to the stopover site had a 15% higher probability of departing the stopover site on the day of arrival than early conspecifics, and females had a 15% higher probability of being a transient than males (Table 3).

**Table 3:** Summary of the estimated probability (±*SE*) that a newly caught individual is a transient (e.g., departs stopover site on the day of capture), and the estimated stopover duration of nontransient individuals calculated from model averaged parameter estimates of top models

Variable	Percent transient (%)	Stopover duration (days)
Breeding		
Southeastern	0 ± 11.0	2.8 ± 0.2
Boreal forest	51.6 ± 13.3	2.6 ± 0.1
Timing		
Early	19.3 ± 18.1	3.8 ± 0.2
Middle	0 ± 18.09	1.7 ± 0.1
Late	34.9 ± 14.6	2.5 ± 0.1
Sex		
Male	8.0 ± 14.5	2.3 ± 0.1
Female	22.7 ± 11.1	3.1 ± 0.2

For individuals that utilized the stopover site beyond the day of arrival, the duration of time spent at the stopover site was influenced most strongly by migratory condition, but also breeding destination (Tables 2 and 3; RI = 1.00, 0.42, respectively). There was a negative relationship between stopover duration and a bird's condition with the average stopover duration increasing by 0.26 ± 0.08 days for each unit reduction in migratory condition, such that a bird arriving with reduced body condition below lean body mass (e.g., condition index = −1.40) would be estimated to stopover for 2.79 days compared to only 1.12 days for a bird arriving with excess fat reserves (e.g., condition index = 3.0). While differences in stopover duration are minimal, warblers in close proximity to their breeding grounds in the southeastern U.S. had longer durations of stopover than warblers migrating to more northern breeding destinations in the boreal forest of Canada (Table 3, Fig. 3b). There was only weak support for females having longer stopover durations than males (RI = 0.17), and birds arriving late to the stopover site, regardless of breeding destination, stopping over for shorter time periods than earlier arriving conspecifics (RI = 0.08; Table 3, Fig. 3b).

## 4 DISCUSSION

Our study demonstrates that migrants adjust their stopover strategies in relation to their time‐schedule and distance to breeding destination, highlighting that strategies of migration should be examined in the context of the annual cycle. By geographically linking an individual captured during spring migration with a breeding destination, we were able to determine an individual's proximity to their breeding destination and whether an individual arrived early or late to the stopover site compared to conspecifics migrating to the same breeding destination. We found that a black‐and‐white warbler's stopover strategy (refueling rate and stopover duration) is not only influenced by a bird's condition upon arrival, but also (1) the distance remaining to a bird's breeding destination, and (2) the timing of arrival at the stopover site, which is largely driven by habitat conditions experienced in the previous phase of the annual cycle (Paxton & Moore, 2015).

Where a bird is in relation to its final breeding destination influenced how an individual utilized the stopover site. Black‐and‐white warblers migrating short distances to breeding destinations in the southeastern U.S. refueled at higher rates and were more likely to stay at the stopover site beyond the day of arrival (i.e., low transient probability) than warblers migrating to more northern breeding destinations in the boreal forest of Canada, despite having arrived at the stopover site in better migratory condition (Paxton & Moore, 2015). Whereas we may expect all migrants to replenish energy reserves after crossing an geographic feature such as the Gulf of Mexico, our results suggest that birds near their breeding destination accumulate additional energy stores, most likely in preparation for breeding (sensu Gudmundsson, Lindstrom, & Thomas, 1991; Sandberg & Moore, 1996). Additionally, birds in close proximity to their breeding grounds may be more likely to stay beyond the day of arrival to assess environmental and phenological conditions at the stopover site, providing an indicator of conditions they are likely to encounter on their breeding grounds (Marra, Francis, Mulvihill, & Moore, 2005; Robson & Barriocanal, 2011; Winkler et al., 2014). Reproductive success is not only tied to optimal timing of arrival on the breeding grounds, particularly for males (Moller, 1994; Norris et al., 2004; Tonra, Marra, & Holberton, 2011), but also a bird's condition (Moore, Smith, et al., 2005). For example, female American redstarts (*Setophaga ruticilla*) and pied flycatchers (*Ficedula hypoleuca*) that arrived at their breeding grounds with surplus fat reserves laid or hatched significantly more eggs (Moore, Smith, et al., 2005). Excess fat reserves when arriving at the breeding grounds can benefit a bird by allowing it to sustain itself against inclement weather encountered during early spring on the breeding grounds (Moller, 1994; Smith & Moore, 2005) or devote more time to territory or mate acquisition, accelerating the start of reproduction (Romero, Soma, O'Reilly, Suydam, & Wingfield, 1997). Given the importance of time and condition on reproductive performance, we would expect strong selective pressures to act on migration strategies to optimize the speed of migration and a bird's migratory condition (Alerstam & Lindstrom, 1990; Jenni & Schaub, 2003). Our findings provide evidence that selection pressures associated with time and condition may change with proximity to breeding destination with the importance of condition potentially playing a stronger role when birds near their breeding destination.

The interaction between selection pressures associated with time and condition en route is also evident when stopover strategies are examined in the context of not only breeding destination, but also a bird's time‐schedule. Black‐and‐white warblers exhibit a chain migration pattern, in which, warblers wintering in the eastern winter range (e.g. Dominican Republic, Jamaica, Puerto Rico) migrate to breeding locations at southern latitudes in eastern North America, while warblers wintering in the western winter range (e.g. Mexico, Belize, Nicaragua) migrate to breeding locations at northern latitudes in the boreal forest of Canada (Marra & Studds, 2013). While the specific winter origin of warblers captured at the stopover site can only be inferred based on migratory connectivity, the timing of black‐and‐white warblers arrival to the stopover site is tightly linked with winter habitat quality, with early arriving birds originating from higher‐quality winter habitat than later arriving conspecifics migrating to the same breeding destination (Paxton & Moore, 2015). However, the migratory condition of black‐and‐white warblers is only diffusely related to a bird's winter habitat quality, regardless of breeding destination (Paxton & Moore, 2015). In this study, we found differences in refueling rates and stopover duration, reflective of differences in migratory condition and timing of arrival at the stopover site, only among black‐and‐white warblers migrating to breeding destinations in the southeastern U.S. Males migrating to the southeastern U.S., whether arriving early or late to the stopover site, had lower migratory condition (Paxton & Moore, 2015) and higher refueling rates than birds arriving during mid migration, suggesting they utilized the stopover site to re‐gain lost energy reserves prior to arrival at their breeding grounds. Yet, birds arriving late to the stopover site were more likely to depart the day of arrival at the stopover site (i.e., transients), and among birds that stopped over at the site, the average duration of stopover for late birds was almost half the time of early conspecifics. Thus, birds arriving late to the stopover site potentially decreased their duration of stopover in order to “catch‐up” with the overall time‐schedule of migration for optimal arrival timing at the breeding grounds. The timing of arrival on the breeding grounds between successful and unsuccessful breeders can vary by only a few days (Tonra et al., 2011). Thus, minimizing the time spent at a stopover site by only one or two days for earlier arrival on the breeding grounds has the potential to positively influence reproductive success, especially if this strategy is employed at multiple stopover sites across a bird's migratory route. Advanced spring conditions encountered by late birds at stopover sites may provide an indicator of optimal conditions along the remaining migration route and on the nearby breeding grounds (Robson & Barriocanal, 2011; Winkler et al., 2014), accelerating departure of late birds from stopover sites. The adjustment of migration speed for late migrants across their migration route is consistent with a recent wood thrush (*Hylocichla mustelina*) study utilizing day‐light level geolocators that found birds departing late from poor‐quality winter habitat compensated for delays with shorter stopover durations (McKinnon et al., 2015; see also Warnock, Takekawa, & Bishop, 2004; Atkinson et al., 2007 for shorebirds). Our results suggest that birds in close proximity to their breeding destination use stopover strategies that optimize condition and/or time depending on their migratory condition and time‐schedule.

In contrast, we found black‐and‐white warblers migrating long distances to breeding destinations in the boreal forest of Canada all had similar stopover strategies that optimized time over condition. Regardless of migratory condition or timing of arrival at the stopover site, black‐and‐white warblers migrating to the boreal forest had consistently below average refueling rates and were more likely to depart the stopover site on the day of arrival (i.e., transient), suggesting that they primarily used the stopover site to rest after nonstop flight across the Gulf of Mexico before quickly resuming migration. Birds arriving early to the stopover site that utilized the stopover site beyond the day of arrival did, however, have slightly longer durations of stopover than later arriving conspecifics, possibly due to the reduced migratory conditions of long‐distance early migrants compared to later arriving conspecifics (Paxton & Moore, 2015). We predicted black‐and‐white warblers migrating to breeding destinations at northern latitudes would exhibit a time minimization strategy of high refueling rates and short stopover durations in order to reduce the overall time spent migrating and the risk of mortality (sensu of Alerstam & Lindstrom, 1990). However, lower migratory conditions (Paxton & Moore, 2015) and reduced refueling rates of warblers migrating to breeding destinations at more northern latitudes, suggests that birds with long distances remaining on migration potentially accelerate the speed of migration by spending less time at stopover sites and carrying minimal fuel loads. This strategy would be beneficial in view of the long distances remaining on migration and the increased flight costs and risk of predation associated with increased fuel loads (Kullberg, Fransson, & Jakobsson, 1996; Lind & Cresswell, 2006). Research at more northern stopover sites would shed light on whether stopover strategies of birds breeding in the boreal forest of Canada change as they approach their breeding destination, consistent with behavior of birds destined to southeastern U.S. breeding sites.

We did not find strong evidence for the utilization of different stopover strategies at the stopover site between males and females, despite likely differences in the stringencies of time pressures between the sexes during migration (Dierschke, Mendel, & Schmaljohann, 2004; Moore, Mabey, & Woodrey, 2003). Female black‐and‐white warblers consistently had higher migratory condition upon arrival to the stopover site than males, regardless of the timing of migration or breeding destination (Paxton & Moore, 2015). Yet, there were minimal differences in refueling rates and stopover duration between the sexes with males tending to have higher refueling rates and shorter durations of stopover than females, consistent with males utilizing a time minimization strategy. Increased sample sizes for the sexes may elucidate whether stronger differences in stopover strategies exist between males and females.

Controlling for yearly and population‐specific differences in the timing of arrival to the stopover site allows for the examination of stopover strategies in the context of both a bird's time‐schedule and distance remaining to a breeding destination. However, the rate at which a migrant refuels and the time spent at a stopover site can also be influenced by extrinsic factors associated with a stopover site (e.g., food availability, weather conditions) that can vary from year‐to‐year. There is growing evidence that migrants can adjust the speed of migration, either delaying or increasing migration speeds, based on phenological and environmental conditions experienced on the wintering grounds or en route (Marra et al., 2005; Robson & Barriocanal, 2011; Tottrup et al., 2008). However, there was little annual variability in the average timing of migration for black‐and‐white warblers, the start of spring, or food availability at the stopover site during the time period of the study. Based on the USA National Phenological Network first bloom spring index (Schwartz, Ault, & Betancourt, 2013), the start of spring at the study site in Cameron Parish, Louisiana consistently began in mid February, except for the delayed bloom during the 2010 El Nino event (day of year for first bloom 2008–2011: 50,47,67,57; USA‐NPN 2017). Likewise, food availability at the stopover site, based on arthropod abundance on the three dominant plant species at the study site, was similar within and among migration seasons. Further, long‐term data at the study site show annual variability in the timing of passage at the stopover site is unrelated to spring resource phenology within wintering areas or stopover regions on the southern Gulf of Mexico (e.g., Yucatan Peninsula), suggesting that any adjustments in the rate of migration most likely occurs north of the study site (Cohen et al., 2015). Thus, the variation in refueling rates and stopover lengths of black‐and‐white warblers at the stopover site was most likely due to differences in stopover strategies within (e.g., early vs. late) and among breeding populations (e.g., southeast and boreal forest) and not driven by seasonal or annual variation in resources and phenology.

Our study provides empirical evidence that a migrant's stopover strategy is not only a function of a bird's current energetic state, but also the distance remaining to breeding destination as well as a bird's time‐schedule, which is tightly linked with habitat conditions experienced during the previous phase of the annual cycle (Paxton & Moore, 2015). Moreover, we show that selection pressures associated with time and condition may change with proximity to breeding destination and a bird's time‐schedule. These findings highlight the importance of incorporating information from other phases of the annual cycle when examining factors known to be important to the success of migration. Without an annual cycle approach, population‐specific difference in en route migration strategies would not have been detected, variation in responses over‐estimated, and stopover strategies for black‐and‐white warblers overall potentially misinterpreted. The ability to link small landbirds to distinct locations over a large geographic area through stable isotopes (Hobson & Wassenaar, 2008), genetic markers (Ruegg et al., 2014, 2017), and light‐weight tracking devices (Bridge et al., 2011) makes it increasingly possible to examine the migratory phase of the annual cycle in the context of other phases of the annual cycle. This achievement is especially important in light of the rapid declines of migratory species compared to nonmigratory species (Wilcove & Wikelski, 2008). Understanding the drivers of declines and implementing effective conservation strategies will require an annual cycle approach.

## CONFLICT OF INTEREST

None declared.

## AUTHOR CONTRIBUTIONS

KLP and FRM conceived and designed the study. KLP conducted statistical analysis and wrote the manuscript. FRM contributed substantially to revisions. All authors gave final approval for publication.

## Appendix 1

### 1.1

Day of the year that black‐and‐white warblers were captured during spring migration 2008 to 2011 on the northern Gulf of Mexico. Box plot whiskers depict the 10th and 90th percentiles and boxes show the 25th and 75th percentiles with the median value indicated; circles represent outliers. Letters above bars indicate years that are significantly different from one another, taking into account breeding destination and sex, based on a Tukey's post‐hoc analysis. Taking into account breeding destination and sex, black‐and‐white warblers arrived on average 6 days earlier in 2011 compared to the three previous years.
